# Exploring the relationship between multiple dimensions of subjective socioeconomic status and self-reported physical and mental health: the mediating role of affect

**DOI:** 10.3389/fpubh.2023.1138367

**Published:** 2023-07-28

**Authors:** Pål Kraft, Brage Kraft

**Affiliations:** ^1^Department of Psychology, Faculty of Social Sciences, University of Oslo, Oslo, Norway; ^2^Department of Psychology, Oslo New University College, Oslo, Norway; ^3^Division of Psychiatry, Diakonhjemmet Hospital, Oslo, Norway

**Keywords:** income, education, subjective socioeconomic status, personal relative deprivation, negative affect, positive affect, self-reported physical health, self-reported mental health

## Abstract

**Introduction:**

This study aimed to investigate the predictive effects of two types of subjective socioeconomic status on self-reported physical and mental health. Specifically, we examined the MacArthur Scale (MacArthur) which measures perceived socioeconomic rank in the society and a novel scale called ComSim, which assessed how participants compared themselves socioeconomically to others coming from a similar socioeconomic background. We also considered the influence of income, education, and personal relative deprivation (PRD) in these analyses. Additionally, we explored whether these effects were mediated through negative and positive affect.

**Methods:**

The data were collected through a cross-sectional, two-wave survey of 294 women and 294 men, with a mean age 41.6 years. Participants were recruited via an online platform.

**Results:**

The results from multivariate regression models revealed that socioeconomic status measured with both the MacArthur Scale and ComSim significantly predicted both self-reported health measures, whereas income and education did not predict any of these measures in the full multivariate models. PRD only predicted self-reported mental health. Mediation analyses showed that negative and positive affect mediated the relationships between socioeconomic status measured by ComSim and self-reported health measures.

**Discussion:**

These findings are discussed in the context of the similarity hypothesis of social comparison theory. The results underscore the importance of considering multiple dimensions when examining socioeconomic health disparities.

## 1. Introduction

The mechanisms that explain the relationship between an individual’s socioeconomic status (SES) and health, are still incompletely understood (1). The materialist explanation posits that poorer health is a consequence of a lack of tangible resources, such as money and education (2, 3). The relativity hypothesis states that health is negatively influenced by the experience of being comparatively low in a socioeconomic hierarchy (4). Additionally, poorer health is associated with an increased perception of unfair social inequality and personal relative deprivation (PRD), which can negatively impact health by causing feelings of anger, frustration and other negative emotions (5–8). In both explanations, negative affect is considered a key mediating mechanism (4, 6, 8, 9). Based on insights derived from social comparison theory and research (10, 11), this paper extends previous research in two ways. First, by applying the similarity principle of social comparison theory, we examined if comparing one’s socioeconomic situation with others coming from a similar socioeconomic background (ComSim) predicted self-reported health above and beyond income, education, socioeconomic status measured with the MacArthur scale, and PRD. Next, considering that people may engage in both upward and downward social comparison which may lead to negative and positive affect, respectively, we simultaneously examined both positive and negative affect as mediators in the relationship between SES and self-reported health.

Research has documented a relationship between income and education and both objective and self-reported health (12–16). For example, lower absolute income may negatively influence health through increased exposure to hazardous wastes and toxins, air pollutants, poor water quality, noise, poor housing quality, unsafe work environments, lower access to adequate health services, and more financial stress (17–20). Education, which is often moderately related to income (21, 22), seems to influence health more indirectly via social and cultural resources, as well as patterns of decision making and behaviors (23–25).

Two observations have indicated that a materialist explanation may not fully explain socioeconomic health inequality. First, income has an effect on health all along the income distribution, an observation known as the socioeconomic health gradient paradox (26). Second, health is also associated with a person’s experience of their relative position in the socioeconomic hierarchy in a community or society (4). Perceived socioeconomic position is associated with longevity, objective health (physical and mental), and many biomarkers (27) and psychological predictors of health (4, 28–30). Perceived socioeconomic position has been found to outperform income and education in predicting self-reported health (4, 27–29, 31, 32). It is assumed that lower perceived socioeconomic position negatively influences health through pathways leading to elevated levels of physical and psychological stress (14, 33, 34).

The most widely applied measure of perceived socioeconomic position is The MacArthur Scale (4), which represents a cognitive assessment of one’s socioeconomic position. However, it is possible that two people may have the same self-perceived socioeconomic position, but different affective reactions to it. To account for such possibility, PRD simultaneously assesses the appraisals that one is unfairly worse off than others, and the subsequent feelings of resentment and anger (5, 35). Callan et al. (5) claimed that PRD better adheres to the similarity hypothesis of social comparison theory (10, 11), stating that comparing oneself with similar others is psychologically more important than comparing oneself to general others in one’s community or society, which is done when measuring subjective SES with the MacArthur scale. Indeed, research shows that PRD tends to outperform income, education and the MacArthur subjective SES scale in predicting self-reported health (5, 36–38).

However, in PRD research, socioeconomic factors are referred to in a rather implicit way. For example, these are two items from the *Personal Relative Deprivation Scale* (PRDS) (39): “I feel deprived when I think about what I have compared to what other people like me have” and “I feel dissatisfied with what I have compared to what other people like me have.” In these items, “have” seems to allude to socioeconomic resources. Nevertheless, it is possible that such implicit wording may, in some participants, prompt perceptions about something other than socioeconomic resources, such as various types of cultural, social, or personal resources. Moreover, PRD does not specify which reference person or group the term “like me” should bring to the participant’s mind. This may be less than optimal if one’s focus is specifically on socioeconomic inequality. The reason is that individuals may have any number of personal and social identity markers by which they describe and compare themselves to others, the psychological salience of which may vary with time and situations (40). Hence, there is some possibility that PRDS items may prompt thoughts about something other than one’s socioeconomic situation, for example the participant’s gender, race, ethnicity, age, sexual orientation, disability, geographic location, personality, abilities, or some other identity marker. The present paper takes this into account by asking participants to explicitly compare themselves based on money, job, education, and social status vis-a-vis others coming from a similar socioeconomic background (ComSim). We examined if subjective SES measured with ComSim predicted self-reported health over and above income, education, subjective SES measured with the MacArthur scale, and PRD.

Finally, we addressed another potential gap in the SES–health literature: the role of affect in mediating the relationship between SES and health. Specifically, research has demonstrated that negative affect plays a key role in mediating the relationship between SES and health (4–6, 8, 9, 30). One possible mechanism is that people engage in upward social comparison, which may foster unfavorable self-evaluations and negative affect (41–44), and ultimately poorer health (5–9, 45). Such a mechanism is all the more likely in modern societies, which are characterized by high social competition (46, 47) and the fact that many people have ample access, via social media, to information about people who are considered to be similar to them in some ways, but who are still richer and more successful. However, in contrast to the focus on negative affect in previous research, a potential role of positive affect remains uninvestigated. This may seem unfortunate, as living in a socioeconomic hierarchy also makes it possible to compare oneself with others who appear to be worse off (41, 48, 49); downward social comparison may represent a source of self-enhancement and positive affect, which is probably what motivates such comparisons (49–53). Some individuals may choose such a strategy when instrumental action to change their objective situation is perceived as difficult or impossible (41, 42, 48, 53, 54). As both negative and positive affect are associated with various health outcomes (55–58), it is unfortunate that previous research has focused exclusively on negative affect in mediating the SES–health relationship (55, 57, 58). Therefore, we examined both negative and positive affect simultaneously as potential mediators of the SES–health relationship.

In sum, we investigated the following hypotheses: (I) Income and education predicts self-reported health. (II) Subjective SES measured with the MacArthur scale predicts self-reported health over and above income and education. (III) PRD predicts self-reported health over and above income, education and subjective SES measured with the MacArthur scale. (IV) Changes in subjective SES over time, as measured with ComSim, predicts self-reported health over and above income, education, PRD, and a static comparison with general others in society (the MacArthur scale). (V) Negative and positive affect simultaneously mediates the relationship between SES measures and self-reported health.

## 2. Materials and methods

### 2.1. Participants and procedure

A two-wave survey design was employed. Data on age, sex, income, education, subjective SES measured with the MacArthur scale, subjective SES measured with ComSim, PRD, and positive and negative affect were collected at T1. Data on self-reported physical and mental health were collected at T2, 3 months later. Participants were recruited via Prolific Academic, an online, crowd-working platform tailored for research, which seems to provide high data quality (59, 60). Participants meeting the following criteria were invited to participate in the study: age 25–60 years (as most people are likely to have finished their education by the age of 25), having United Kingdom/British citizenship (to ensure homogenous reporting of education), and English as their first language (to ensure adequate comprehension of the questions). At T1, participants accessed a link to the online survey, read the study information, and were routed to an online Qualtrics questionnaire. At T2, participants were contacted by email containing a link to the second Qualtrics survey. To ensure sufficient power to detect a small mediation effect with 0.8 power and estimated 75% retention of participants from T1 to T2, we aimed at recruiting at least 670 participants, balanced between the sexes, at T1.

### 2.2. Measures

*Self-reported physical health* (SRPH) was measured by: “How would you rate your physical health at the present time?” *Self-reported mental health* (SRMH) was measured by “How would you rate your mental health at the present time?” Both measures were reported on five-point scale ranging from *very bad* to *very good*.

*Positive and negative affect* were assessed using the positive and negative affect schedule (PANAS) (61). In the Positive and Negative Affect Schedule (PANAS), positive affect refers to the extent to which a person experiences positive emotions such as joy, enthusiasm, and alertness. It reflects the degree to which an individual feels enthusiastic, excited, active, and interested in their surroundings. On the other hand, negative affect refers to the extent to which a person experiences negative emotions such as fear, anger, and sadness. It reflects the degree to which an individual feels nervous, upset, distressed, or irritable. The PANAS is a widely used tool to measure both positive and negative affect and has been used in research to assess the impact of different factors on individuals’ emotional states. PANAS measures positive and negative affect using two 10-item scales. We used the following time instruction: “Indicate to what extent you have felt this way during the past month.” Each item was responded to on a five-point scale, ranging from *very little* to *a lot*.

*Income* personal income after tax was reported in 12 categories ranging from <£10,000 to >£150,000 (for details see the Results section).

*Education* level was assessed using the question: “Which of these is the highest level of education you have completed?” Participants were provided with 12 options, ranging from no formal qualifications to a doctorate degree (for details see the Results section).

*The MacArthur Scale of Subjective Social Status—Adult Version* (26) was used to assess perceived socioeconomic position. Respondents viewed a drawing of a ladder with 10 rungs and a corresponding text reading: “This ladder represents where people stand in society. At the top of the ladder are people who are the best-off, those who have the most money, most education, and best jobs. At the bottom are people who are the worst-off, those who have the least money, least education, worst jobs, or no job. Please place an ‘X’ on the rung that best represents where you think you stand on the ladder.”

*PRD* was assessed by Callan et al. (39) five-item *personal relative deprivation scale* (PRDS), which taps people’s perceptions and emotions associated with comparing their outcomes to the outcomes of others considered to be like themselves. Items were rated on a six-point scale ranging from *strongly disagree* to *strongly agree*.

*ComSim* was developed specifically for this study and was measured by four items constructed to assess how people compared their current socioeconomic situation with others coming from a similar socioeconomic background. Respondents were presented with an introductory text reading: “People come from different social classes or socioeconomic backgrounds. This reflects how much money your parents had; how much education your parents had; your parent’s social position; your housing conditions when you were a child; the schools you attended; your place of living as a child. When responding to the statements below, think about such elements in your socioeconomic background, that is, where you come from socioeconomically.” Then, participants responded to four statements: “Compared to other people coming from a similar socioeconomic background, my current financial situation is quite good”; “Compared to other people coming from a similar socioeconomic background, my current educational situation is quite good”; “Compared to other people coming from a similar socioeconomic background, I have been quite successful in work-life”; and “Compared to other people coming from a similar socioeconomic background, I think my current socioeconomic position is quite good.” The items were responded to on a seven-point scale ranging from *strongly disagree* to *strongly agree*. A principal component factor analysis (varimax rotation) was performed on the four items and produced a one-factor solution, which explained 78.9% of the inter-item variance (Eigenvalue = 3.15). The one-factor solution was confirmed in a confirmatory factor analysis using SPSS Amos (χ^2^ = 15.202; df = 2; *p* < 0.001; CFI = 0.992; GFI = 0.988). The scale showed acceptable internal consistency reliability (Cronbach’s alpha = 0.85, Table 1).

**Table 1:** Descriptive statistics (*N* = 588).

Variables	Possible	Min range	Max	Mean	Std	α
Education	1–7	1.00	7.00	4.24	1.39	-
Income	1–12	1.00	12.00	4.70	2.49	-
MacArthur	1–10	1.00	10.00	5.22	1.65	-
PRD	1–6	1.00	5.60	5.07	1.32	0.85
ComSim	1–7	1.00	7.00	3.00	0.57	0.91
Negative affect	1–5	1.00	5.00	2.98	0.82	0.91
Positive affect	1–5	1.00	5.00	1.93	0.73	0.92
SRPH	1–5	1.00	5.00	3.55	0.82	-
SRMH	1–5	1.00	5.00	3.53	0.94	-

MacArthur, MacArthur scale of subjective socioeconomic status; PRD, personal relative deprivation; ComSim, comparing oneself socioeconomically to similar others; SRPH, self-reported physical health; and SRMH, self-reported mental health.

### 2.3. Analysis

We used SPSS version 29 to conduct all analyses. To begin, we provided descriptive statistics and examined the bivariate correlations between variables. Next, we performed several linear multiple regression analyses (using the enter method) to test hypotheses I, II, III, and IV. SPSS Amos 29 was used for confirmatory factor analysis. In order to assess mediation (hypothesis V), we utilized the PROCESS macro for SPSS (62) and applied model 4. This analysis calculates the direct, indirect, and total effects of an independent variable on a dependent variable using 5,000 bootstrap samples. Our reporting of the results included standardized regression coefficients (path coefficients), standard errors (SE), 95% confidence intervals (CI) of the regression coefficients, and t-and *p*-values. To determine if an indirect effect was significant, we looked for a 95% CI interval of the effect that did not include zero, indicating significant mediation.

## 3. Results

We stopped recruitment when we reached 679 participants, 339 men and 340 women. Of these, 588 took part in the T2 data collection, reaching a retention rate of 86.6%. The final T2 sample included 294 women and 294 men; with a mean age of 41.6 years (SD = 9.9 years). No differences in T1 variables were observed between completers and drop-outs of the T2 survey.

To balance the variable distribution, income was recoded into six categories: (1) < £10,000 (*n* = 131), (2) £10,000–£19,999 (*n* = 129), (3) £20,000–£29,999 (*n* = 151), (4) £30,000–£39,999 (*n* = 93), (5) £40,000–£49,999 (*n* = 55), and (6) ≥ £50,000 (*n* = 29). For the same reason, education was recoded into five groups: (1) no formal education/secondary education (e.g., GED/GCSE; *n* = 93), (2) high school diploma/A-levels (*n* = 103), (3) technical/community college (*n* = 71), (4) undergraduate degree (BA/BSc/other; *n* = 223), and (5) graduate degree (MA/MSc/MPhil/other)/doctoral degree (*n* = 98). Table 1 presents descriptive statistics.

As shown in Table 2, income and education were moderately correlated (*r* = 0.29, *p* < 0.01), and were hence kept separate for all analyses. Education and income were substantially correlated with MacArthur, PRD, and ComSim, with higher correlations seen for income. Education positively correlated with SRPH (*r* = 13, *p* < 0.001), but not with SRMH. Income positively correlated with SRPH (*r* = 19, *p* < 0.001) and SRMH (*r* = 0.18, *p* < 0.001). Subjective SES measured with the MacArthur scale positively correlated with SRPH (*r* = 30, *p* < 0.001) and SRMH (*r* = 0.33, *p* < 0.001). Notably, the MacArthur scale correlated with SRPH (*r* = 0.32, 0.26, and 0.20) and SRMH (*r* = 0.34, 0.30, and 0.19) within all income groups (low, medium, and high, respectively; *p* < 0.001). PRD negatively correlated with the MacArthur scale (*r* = −0.43, *p* < 0.001), SRPH (*r* = −0.23, *p* < 0.001), and SRMH (*r* = −0.39, *p* < 0.001). Correlation analyses within the three income groups showed that PRD negatively correlated (*p* < 0.001) with SRPH (*r* = −0.18, −0.20, and − 0.22) and SRMH (*r* = −0.40, −0.35, and − 0.34) within the three income groups (low, medium, and high, respectively). Subjective SES measured with ComSim negatively correlated with PRD (*r* = −0.60, *p* < 0.001), but positively with SRPH (*r* = 0.30, *p* < 0.001) and SRMH (*r* = 0.40, *p* < 0.001). Correlation analyses within the three income groups showed that ComSim correlated (*p* < 0.001) with SRPH (*r* = 0.33, 0.21, and 0.26) and SRMH (*r* = 0.46, 0.34, and 0.26) in all income layers (low, medium, and high). Finally, as shown in Table 2, negative affect negatively correlated with subjective SES measured with the MacArthur scale (*r* = −0.21, *p* < 0.001), subjective SES measured with ComSim (*r* = −0.30, *p* < 0.001), SRPH (*r* = −0.28, *p* < 0.001), and SRMH (*r* = −0.54, *p* < 0.001). Positive affect positively correlated positively with the MacArthur scale (*r* = 0.30, *p* < 0.001), the ComSim scale (*r* = 0.43, *p* < 0.001), SRPH (*r* = 0.38, *p* < 0.001), and SRMH (*r* = 0.52, *p* < 0.001).

**Table 2:** Pearson’s correlation (*r*) between variables (*N* = 588).

S.no	Variables	1.	2.	3.	4.	5.	6.	7.	8.	9.
1.	Education		0.29^**^	0.30^**^	−0.10^*^	0.18^**^	−0.04	0.08	0.13^**^	0.06
2.	Income			0.51^**^	−0.30^**^	0.42^**^	−0.13^**^	0.23^**^	0.19^**^	0.18^**^
3.	MacArthur				−0.43^**^	0.55^**^	−0.21^**^	0.30^**^	0.30^**^	0.33^**^
4.	PRD					−0.60^**^	0.33^**^	−0.40^**^	−0.23^**^	−0.39^**^
5.	ComSim						−0.30^**^	0.43^**^	0.30^**^	0.40^**^
6.	Negative affect							−0.35^**^	−0.28^**^	−0.54^**^
7.	Positive affect								0.38^**^	0.52^**^
8.	SRPH									0.53^**^
9.	SRMH									

MacArthur, MacArthur scale of subjective socioeconomic status; PRD, personal relative deprivation; ComSim, comparing oneself socioeconomically to similar others; SRPH, self-reported physical health; SRMH, self-reported mental health.

* *p* < 0.05 (two-tailed tests).

** *p* < 0.01 (two-tailed tests).

### 3.1. Hypothesis I: income and education will predict self-reported health

In multivariate regression, income predicted SRPH (β *= 0*.17, *p* < 0.001) and SRMH (β *= 0*.17, *p* < 0.001), whereas education did not predict any of the health measures (Table 3, Model 1).

**Table 3:** Hierarchical regressions of associations between various measures of SES and self-reported health (standardized beta-coefficients; *N* = 588).

	Dependent variables	
	SRPH	SRMH
Model 1		
Income	0.17^**^	0.17^**^
Education	0.08	0.01
*R* ^2^	0.04	0.03
∆*F*	12.94^**^	9.24
Model 2		
Income	0.04	0.02
Education	0.03	−0.05
MacArthur	0.27^**^	0.33^**^
*R* ^2^	0.09	0.11
∆R^2^	0.05	0.08
∆*F*	33.51^**^	51.44^**^
Model 3		
Income	0.03	−0.02
Education	0.04	−0.03
MacArthur	0.22^**^	0.21^**^
PRD	−0.12^**^	−0.31^**^
*R* ^2^	0.11	0.19
∆*R*^2^	0.01	0.08
∆*F*	7.94^**^	54.43^**^
Model 4		
Income	0.01	−0.05
Education	0.04	−0.03
MacArthur	0.17^**^	0.15^**^
PRD	−0.05	−0.22^**^
ComSim	0.17^**^	0.21^**^
*R* ^2^	0.12	0.21
∆*R*^2^	0.02	0.02
∆*F*	9.79^**^	15.95^**^

^*^p < 0.05, ^**^p < 0.01. MacArthur, MacArthur scale of subjective socioeconomic status; PRD, personal relative deprivation; ComSim, comparing oneself socioeconomically to similar others; SRPH, self-reported physical health; SRMH, self-reported mental health.

### 3.2. Hypothesis II: subjective SES measured with the MacArthur scale predicts self-reported health over and above income and education

As shown in Table 3 (Model 2), subjective SES measured with the MacArthur scale predicted SRPH (β *= 0*.27, *p* < 0.001) and SRMH (β *= 0*.33, *p* < 0.001), whereas income and education did not predict health measures in these multivariate regression models. The inclusion of the MacArthur scale caused a significant increase in *R*^2^ (∆*F* = 33.51 and 51.44, respectively, both *p* < 0.001).

### 3.3. Hypothesis III: PRD predicts self-reported health over and above income, education and subjective SES measured with the MacArthur scale

In the multivariate regression models (Table 3, Model 3), the MacArthur scale and PRD predicted SRPH (β *= 0*.22 and − 0.12) and SRMH (β *= 0.*21 and − 0.31), whereas income and education did not predict the health measures. The inclusion of PRD caused a significant increase in *R*^2^ (∆*F* = 7.94 and 54.43, respectively, both *p* < 0.001).

### 3.4. Hypothesis IV: changes in subjective SES over time, as measured with ComSim, predicts self-reported health over and above income, education, PRD and a static comparison with general others in society (the MacArthur scale).

In multivariate regression models (Table 3, Model 4), subjective SES measured with ComSim significantly predicted SRPH and SRMH (β *=* 0.17 and 0.21), as did subjective SES measured with the MacArthur scale (β *=* 0.17 and 0.15). PRD only predicted SRMH in such models (β *=* −0.22). Income and education did not predict self-reported health in these models. The inclusion of ComSim caused a significant increase in *R*^2^ (∆*F* = 9.79 and 15.95, respectively, both *p* < 0.001).

### 3.5. Hypothesis V: negative and positive affect will mediate the relationship between SES and self-reported health

**Figure 1:**
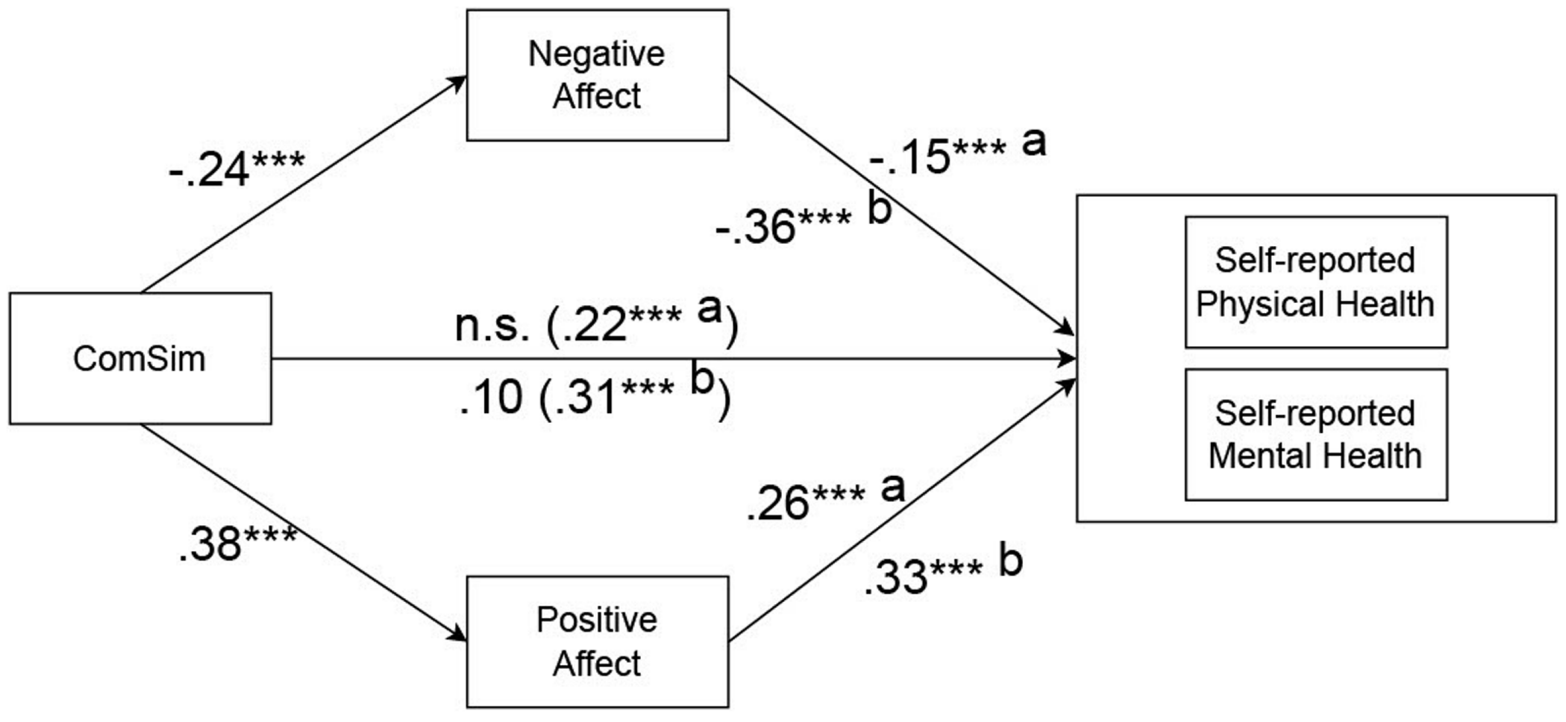
Standardized regression coefficients for the relationship between ComSim and self-reported health measures as mediated by negative and positive affect. Total effects in parentheses; a = effects on self-reported physical health; b = effects on self-reported mental health. Age, sex, income, education, and MacArthur were entered as covariates.

For subjective SES measured with the MacArthur and ComSim scales, respectively, mediation analyses were carried out to test the mediation hypotheses. Mediation via negative and positive affect was tested simultaneously. Age, sex, income, education, and the ComSim and MacArthur scales, respectively, were entered as co-variates. Results are shown in Tables 4, 5 and in Figure 1. Regarding predicting SRPH (Table 4), the total effect of subjective SES measured with the MacArthur scale was 0.19, direct effect was 0.16, whereas the indirect effects via negative and positive affect were non-significant. Regarding SRMH (Table 4), the total effect of MacArthur scale was 0.16; the direct effect was 0.11, the indirect effects mediated via negative and positive affect were non-significant. Table 5 shows that the total effect of subjective SES measured with the ComSim scale in predicting SRPH was 0.22, the direct effect was non-significant, and the indirect effects were 0.04 via negative affect and 0.10 via positive affect. Approximately 63.4% of the total effect of the ComSim scale on SRPH was mediated via negative and positive affect. Regarding SRMH (Table 5), the total effect of the ComSim scale was 0.31, the direct effect was 0.10, and the indirect effects mediated via negative and positive affect were 0.09 and 0.12, respectively. Approximately 67.7% of the total effect of the ComSim scale on SRMH was mediated via negative and positive affect.

**Table 4:** Process macro mediation analyses: negative and positive affect as mediators between MacArthur and self-reported health.

	*b*	*SE*	*t*	*p*	*95% CI*
Predicting self-reported physical health					
MacArthur ➔ Negative affect	−0.05	0.05	−0.91	n.s.	−0.14–0.05
MacArthur ➔ Positive affect	0.09	0.05	1.95	n.s.	−0.01–0.19
MacArthur ➔ SRPH	0.16	0.05	3.46	<0.001	0.07–0.26
Negative affect ➔ SRPH	−0.15	0.04	−3.77	<0.001	−0.24 to −0.08
Positive affect ➔ SRPH	0.26	0.04	6.19	<0.001	0.18–0.35
*Effects*					
Direct	0.16	0.05	3.46	<0.001	0.07–0.26
Indirect effect 1 negative affect^*^	0.01	0.01			−0.01–0.02
Indirect effect via positive affect^*^	0.02	0.01			0.00–0.05
Predicting self-reported mental health					
MacArthur ➔ Negative affect	−0.05	0.05	−0.91	n.s.	−0.14–0.05
MacArthur ➔ Positive affect	0.09	0.05	1.95	n.s.	0.00–0.19
MacArthur ➔ SRMH	0.11	0.04	2.81	<0.01	0.03–0.19
Negative affect ➔ SRMH	−0.36	0.03	−10.40	<0.001	−0.43 to −0.29
Positive affect ➔ SRMH	0.33	0.04	9.26	<0.001	0.26–0.40
*Effects*					
Direct	0.11	0.04	2.81	<0.01	0.03–0.19
Total indirect effect via negative affect^*^	0.02	0.02			−0.02–0.05
Total indirect effect via positive affect^*^	0.03	0.02			0.00–0.07

Standardized regression coefficients based on 5,000 bootstrap samples (*N* = 588). Age, sex, income, education, and ComSim were entered as co-variates. ^*^Boots SE and CI.

**Table 5:** Process macro mediation analyses: negative and positive affect as mediators between ComSim and self-reported health.

	*b*	*SE*	*t*	*p*	*95% CI*
Predicting self-reported physical health					
ComSim ➔ Negative affect	−0.24	0.05	−5.04	<0.001	−0.33 to −0.15
ComSim ➔ Positive affect	0.38	0.05	8.11	<0.001	0.28–0.47
ComSim ➔ SRPH	0.08	0.05	1.62	n.s.	−0.02–0.17
Negative affect ➔ SRPH	-0.15	0.04	−3.77	<0.001	−0.24 to −0.07
Positive affect ➔ SRPH	0.26	0.04	6.19	<0.001	0.18–0.35
*Effects*					
Direct	0.08	0.05	1.62	n.s.	−0.02–0.17
Indirect effect via negative affect^*^	0.04	0.01			0.02–0.06
Indirect effect via positive affect^*^	0.10	0.02			0.06–0.14
Predicting self-reported mental health					
ComSim ➔ Negative affect	−0.24	0.05	−5.04	<0.001	−0.33 to −0.15
ComSim ➔ Positive affect	0.38	0.05	8.12	<0.001	0.28–0.47
ComSim ➔ SRMH	0.10	0.04	2.36	<0.05	0.02–0.18
Negative affect ➔ SRMH	−0.36	0.04	−10.40	<0.001	−0.43 to −0.29
Positive affect ➔ SRMH	0.33	0.04	9.26	<0.001	0.26–0.40
*Effects*					
Direct	0.10	0.04	2.36	<0.05	0.02–0.18
Total indirect effect via negative affect^*^	0.09	0.02			0.05–0.13
Total indirect effect via positive affect^*^	0.12	0.02			0.09–0.17

Standardized regression coefficients based on 5,000 bootstrap samples (*N* = 588). Age, sex, income, education, and MacArthur were entered as co-variates. ^*^Boots SE and CI.

## 4. Discussion

The primary objective of this study was to examine the relationship between socioeconomic status (SES) and self-reported health, through the testing of five hypotheses. Encouragingly, our findings provided support for all of these hypotheses. Specifically, we observed significant correlations between income, education, and self-reported health. Notably, our regression analysis revealed that income held greater significance compared to education in predicting self-reported health. Secondly, our investigation revealed that the MacArthur scale of subjective SES demonstrated predictive power in relation to self-reported health, even after accounting for the influence of income and education. Thirdly, our results indicated that PRD emerged as a significant predictor of self-reported health, controlling for the effects of income, education, and the MacArthur scale of subjective SES. Fourthly, our findings demonstrated that changes in socioeconomic position as compared to others, ComSim, maintained its predictive capacity for self-reported health, even when accounting for the influences of income, education, the MacArthur scale of subjective SES, and PRD. Finally, through mediation analyses, we discovered that negative and positive affect mediated the association between the ComSim scale and self-reported health. It is important to note that in the case of the MacArthur subjective SES scale, negative and positive affect did not mediate its relationship with self-reported health.

In the final multivariate models (Table 3, model 4), both the MacArthur and ComSim scales of subjective SES emerged as predictors of self-reported physical and mental health, whereas income and education did not demonstrate significant effects. This observation suggests that the MacArthur and ComSim scales of subjective SES capture distinct forms of social comparison. The MacArthur scale of subjective SES encompasses static comparisons with individuals in the broader society, while the ComSim scale involves comparisons with others coming from similar socioeconomic backgrounds. These findings align with the relativity hypothesis of socioeconomic health inequality, shedding light on the mechanisms underlying this phenomenon. Moreover, our results contribute to the understanding of socioeconomic health inequality and the gradient paradox, providing valuable insights into the complexities of these phenomena.

Engaging in favorable comparisons with individuals from a similar socioeconomic background was associated with higher levels of positive affect, lower levels of negative affect, and better self-reported physical and mental health. These findings align with the similarity hypothesis of social comparison theory, which suggests that comparisons with similar others hold particular psychological significance (11).

One possible explanation for these results is that comparisons with individuals with whom one shares direct experiences and close social ties provide valuable diagnostic information about the self. Such information holds significance for self-evaluations and overall well-being (10, 63). Notably, measuring subjective SES with the MacArthur scale also predicted self-reported health alongside measuring subjective SES with ComSim in our multivariate models (Table 3, Model 4). This underscores the importance of the MacArthur scale for understanding health outcomes, which is consistent with prior research (4, 27, 29, 31, 32, 64, 65). In summary, this study contributes to our understanding by highlighting how two distinct types of social comparison predict self-reported physical and mental health. Both comparing oneself socioeconomically to others coming from a similar socioeconomic background (ComSim) and comparing oneself socioeconomically to general others in the society (the MacArthur scale) represent measures of social comparison, albeit in different social systems.

Various explanations have been proposed to explain the association between perceived socioeconomic status (SES) and health. One such explanation is the averaging hypothesis (65, 66), which suggests that objective SES indicators, such as income and education, often capture limited static and current socioeconomic resources and adversities that may influence health outcomes. In contrast, an individual’s perceptions, as assessed by measures like the MacArthur and ComSim scales of subjective SES, may reflect a cognitive average encompassing a broader range of socioeconomic indicators related to past, present, and future circumstances that influence health but are not explicitly measured in objective indicators (67).

Consequently, perceptions of SES, when associated with this broader set of unmeasured indicators of tangible socioeconomic resources, may explain how unmeasured objective SES variables relate to health. Multiple research findings support this explanation. Firstly, studies have shown that socioeconomic status measured with the MacArthur scale partially mediates the effect of income and education (28, 68). Secondly, research reports indicate that the correlation between the MacArthur scale and a sum score of objective SES increases with the inclusion of a greater number of explicitly assessed objective SES indicators (4, 69). Finally, studies highlight a substantial conceptual and empirical overlap between subjective SES measured with the MacArthur scale and objective SES indicators such as income and education (70).

Despite the aforementioned observations, both from this study and previous research (28, 71) it appears likely that perceived socioeconomic status exerts a distinct and unique influence on health, which is theoretically and empirically distinct from the effects captured by tangible, objective socioeconomic resources. This study suggests the existence of two mechanisms through which this occurs: a static comparison with general others in society, as measured with the MacArthur scale, and changes in socioeconomic position over time compared to similar others, as measured with ComSim.

These social comparison processes are likely to be underpinned by unique psychological mechanisms. Perceived lower societal position is associated with adverse health outcomes due to elevated levels of chronic physical and psychological stress arising from living in circumstances characterized by increased unpredictability, uncontrollability, threats, adversities, lack of social network and support, and limited protection, personal control, power, popularity, and future opportunities (4, 19, 23, 24, 33, 34, 70, 72–78). On the other hand, ComSim may primarily reflect perceptions of favorable or unfavorable performance compared to similar others, which is likely to be significant for self-evaluations (41–43, 79). This reasoning aligns with recent research in health sociology, which has indicated that both actual and perceived social mobility are associated with health and well-being (80–88).

The impact of subjective SES measured with ComSim on self-reported health measures was found to be mediated by both negative and positive affect simultaneously (Figure 1). In contrast, the predictive effects of subjective SES measured with the MacArthur scale were not mediated by either negative or positive affect. This novel finding extends previous research. First, it underscores the results of the regression analyses indicating that the ComSim scale was more important than the MacArthur scale in predicting self-reported health. Second, it extends previous research which has primarily focused on the mediating role of negative affect in the relationship between socioeconomic status (SES) and health. However, the potential role of positive affect in this context has largely remained unexplored (4–6, 8, 9, 30). Our finding is consistent with social comparison theory and prior research, indicating that individuals may engage in both upward and downward social comparisons, which can impact negative and positive affect, respectively (41–43, 50, 53, 63, 79, 89, 90). Furthermore, research indicates that negative affect tends to be more prevalent among individuals belonging to lower SES groups (6–9, 91), positive affect is more prevalent in higher SES groups (92–95). Lastly, these findings are in line with previous research demonstrating the association of both negative and positive affect with various health outcomes, health behaviors, as well as physiological and psychological risk factors and biomarkers (55–58, 96–101).

.Although the correlational design of this study precludes making causal conclusions, we can speculate about potential causal pathways that could elucidate the relationship between subjective SES measured with the ComSim scale and self-reported health. The results suggest that one plausible pathway involves social comparison and affect. Individuals who perceive lower socioeconomic achievements compared to similar others may engage in upward social comparison, which could contribute to elevated levels of negative affect and reduced levels of positive affect, ultimately negatively impacting their health. Conversely, individuals with higher relative socioeconomic achievements may engage in downward social comparison, leading to lower levels of negative affect and higher levels of positive affect, potentially benefiting their health. To establish causal relationships and validate these proposed psychological mechanisms, further research employing experimental designs or longitudinal data is necessary. If future investigations support these pathways, they could offer valuable insights into the dynamics of socioeconomic health inequality and shed light on the paradox of the socioeconomic health gradient.

Significantly, PRD emerged as a predictor of both self-reported physical and mental health, over and above the effects of income, education, and the MacArthur scale (Table 3, model 3). However, in the full multivariate model, PRD only predicted self-reported mental health, while subjective SES measured with the MacArthur and ComSim scales continued to predict both self-reported health measures (Table 3, model 4). These findings deviate from prior research, which has often indicated that PRD tends to outperform the MacArthur scale in predicting self-reported health outcomes (5, 36–38, 102–104). Furthermore, the results suggest that PRD showed a stronger association with self-reported mental health compared to physical health (Table 2). Given these findings, it may be beneficial to further examine the PRD instruments used in the study. For instance, one sample item from the PRDS scale is as follows: “I feel deprived when I think about what I have compared to what other people like me have.” It is important to note that potential issues may arise with such items, as individuals are often not highly accurate in discerning the exact causes of their negative affect (105, 106). The question arises as to whether the increased resentment and anger experienced by individuals with lower SES are solely attributable to their lower position in the socioeconomic hierarchy or if other factors contribute to these emotions. Additionally, it is worth considering whether the associations between PRD, affect, and self-reported mental health reflect negative affectivity or negative automatic thoughts regarding the self. This could potentially explain why PRD predicted self-reported mental health but not physical health in the full multivariate model. Such reasoning suggests that perceptions of socioeconomic position and negative affect should be viewed as conceptually and empirically distinct constructs. Experimental research has provided evidence that subjective socioeconomic position (as measured by the MacArthur scale in that particular study) and negative affect are related, yet remain distinct variables (30). Lastly, it is important to note that certain studies propose that PRD may serve as a precursor of poorer mental health outcomes (5), others have highlighted the possibility of a reverse relationship (107).

This study is subject to several limitations. Firstly, while data on independent and dependent variables were collected at different time points, we did not examine changes in these variables over time. As a result, we were unable to establish causal or temporal relationships using fixed effect or cross-lagged panel models. However, it should be noted that longitudinal designs may be constrained by the limited short-and medium-term variability in SES measures. Secondly, although self-reported single-item indicators of physical and mental health have demonstrated predictive value for mortality and morbidity, as well as high test–retest reliability (29, 108–113), social desirability and recall bias may interfere with participants’ reports and the authenticity of such data (114). Thirdly, it is essential to consider that measures of perceived socioeconomic position, affect, and self-reported health might exhibit covariance due to common method variance, reporting bias, or shared measurement of a common trait, such as negative affectivity. These factors could potentially influence the observed associations between the variables of interest.

## 5. Conclusion

In conclusion, the findings of this study demonstrate that subjective SES measured with both the MacArthur scale and the ComSim scale play significant roles in predicting self-reported physical and mental health, even after accounting for income, education, and PRD. These results indicate that two distinct social comparison processes contribute to understanding socioeconomic health inequality and the gradient paradox: a static comparison with general others in the society and socioeconomic changes in position over time as compared to similar others. Moreover, the simultaneous partial mediation of positive and negative affect between the ComSim scale of subjective SES and self-reported health measures aligns with social comparison theory, particularly the similarity hypothesis. The combined importance of subjective SES as measured with both the MacArthur scale and the ComSim scale in predicting self-reported health provides support for the relativity hypothesis of socioeconomic health inequality.

## Data availability statement

The raw data supporting the conclusions of this article will be made available by the authors, without undue reservation.

## Ethics statement

The studies involving human participants were reviewed and approved by Ethics Committee, Department of Psychology, University of Oslo, Norway, ref. number: 16639255. The patients/participants provided their written informed consent to participate in this study.

## Author contributions

PK and BK have made a substantial, direct and intellectual contribution to the work, and approved it for publication.

## Funding

Data collection was funded by a grant from the Department of Psychology, University of Oslo, Norway.

## Conflict of interest

The authors declare that the research was conducted in the absence of any commercial or financial relationships that could be construed as a potential conflict of interest.

## Publisher’s note

All claims expressed in this article are solely those of the authors and do not necessarily represent those of their affiliated organizations, or those of the publisher, the editors and the reviewers. Any product that may be evaluated in this article, or claim that may be made by its manufacturer, is not guaranteed or endorsed by the publisher.
